# P157: Face-touching: a frequent habit for self-inoculation of transmissible infections?

**DOI:** 10.1186/2047-2994-2-S1-P157

**Published:** 2013-06-20

**Authors:** A Kwok, M-L McLaws

**Affiliations:** 1 UNSW Medicine, The University of New South Wales, Sydney, Australia

## Introduction

Touching the mucous membranes of the nose and mouth is a potential for transmission and acquisition of a range of infections. Infection may be spread to others after inoculating ones own hands during face-touching or infections may be acquired via contaminated hands after face-touching.

## Objectives

To investigate the prevalence of face-touching behaviour in medical students.

## Methods

All fifth year UNSW medical students who had completed 4 hours of infection control education in the prior year attending a lecture theatre for a 60min lecture were invited to participate in a video recording for a behaviour observational study. To eliminate bias students were blinded from the aim of the study. Consented students were instructed to sit on one side of the lecture theatre where video recorder was set up. University Ethics approval was obtained. Two researchers observed video tapes independently and tallied the frequency of hand-to-face contacts for each participant using a standardised sheet to record the region (nose, mouth, eye and non-mucous membrane regions) and frequency of each of these regions.

## Results

All 29 students touched their face at least once. 90% (26/29) touched a mucous membrane on the face at least once during 60min of observation. Out of the 2346 touches observed, 1175 were to a non-mucous membrane region and 1171 were touches to nose, mouth or eye regions, with an average 45 mucous membrane touches per student over the 60 mins period (median 29, min 4, max 153). Touching the mouth was the most frequent region at 372 touches, followed by the nose 318 touches and eyes 273 touches. The duration for mouth touching ranged from 1sec to 12sec (median 1sec, mean 2sec), the duration for nose touching ranged from 1sec to 10sec (median <1sec, mean 1sec), eye touching ranged from 1sec to 5sec (median <1sec, mean 1sec).

## Conclusion

The greatest shedding of virus in the community occurs during the prodromal stage of influenza usually 3 days before symptoms and signs. During the prodromal period the prevalent behaviour of face-touching provides the opportunity for acquisition and transmission of infectious material. During public health campaigns to educate and alter the community about reducing their risk of transmission and acquisition the campaign should also focus on modifying our unconscious preening behaviour.

## Disclosure of interest

None declared

